# Simultaneous expression of ClopHensor and SLC26A3 reveals the nature of endogenous oxalate transport in CHO cells

**DOI:** 10.1242/bio.041665

**Published:** 2019-03-05

**Authors:** Teresa Wasiluk, Mina Roueinfar, Kayla Hiryak, Maria Torsiello, Alexander Miner, Jennifer Lee, Michael Venditto, William Terzaghi, Del Lucent, Adam L. VanWert

**Affiliations:** 1 Department of Biology, College of Science and Engineering, Wilkes University, Wilkes-Barre, PA 18766, USA; 2 Department of Electrical Engineering and Physics, College of Science and Engineering, Wilkes University, Wilkes-Barre, PA 18766, USA; 3 Department of Pharmaceutical Sciences, Nesbitt School of Pharmacy, Wilkes University, Wilkes-Barre, PA 18766, USA

**Keywords:** ClopHensor, Oxalate, Chinese hamster ovary, SLC26A3, DRA, CHO, Niflumic acid

## Abstract

ClopHensor, a fluorescent fusion protein, is a dual function biosensor that has been utilized as a tool for the simultaneous measurement of intracellular chloride and pH in cells. ClopHensor has traditionally been used in conjunction with fluorescence microscopy for single cell measurements. Here, we present a promising multi-well format advancement for the use of ClopHensor as a potential high-throughput method capable of measuring fluorescence signal intensity across a well of confluent cells with highly reproducible results. Using this system, we gained mechanistic insight into an endogenous oxalate transporter in Chinese hamster ovary (CHO) cells expressing ClopHensor and the human chloride transporter, SLC26A3. SLC26A3, a known anion exchanger, has been proposed to play a role in colonic oxalate absorption in humans. Our attempt to study the role of SLC26A3 in oxalate transport revealed the presence of an endogenous oxalate transporter in CHO cells. This transporter was strongly inhibited by niflumate, and exhibited clear saturability. Use of ClopHensor in a multi-well cell assay allowed us to quickly demonstrate that the endogenous oxalate transporter was unable to exchange chloride for bicarbonate, unlike SLC26A3.

## Abstract (precis)

**Summary:** We expressed the fluorescent fusion protein, ClopHensor, for the first time in a multi-well format to assess for bicarbonate and chloride exchange by an endogenous oxalate transporter in CHO cells.

## INTRODUCTION

The fluorescent fusion protein, ClopHensor, has previously been reported as a promising tool for simultaneous measurement of intracellular chloride and pH in live cells (Arosio et al., 2010; Mukhtarov et al., 2013). This sensor has a modified green fluorescent protein, E^2^GFP, whose green fluorescence is sensitive to pH, and both green and cyan fluorescence are sensitive to chloride concentration (Arosio et al., 2007). Measuring the green-to-cyan fluorescence ratio precludes the influence of chloride on pH measurement, as chloride affects both signals equally, whereas pH affects only the green signal. This has been described as static quenching, wherein chloride binding to E^2^GFP completely inhibits fluorescence and thus prevents alterations in the ratiometric measurement of pH (Arosio et al., 2010). ClopHensor also possesses a red fluorescent protein, monomeric DsRed, whose signal intensity is not affected by pH or chloride. Therefore, after constructing chloride standard curves at different pH values, one can measure the absolute chloride concentration using the appropriate chloride-pH curve, dictated by the pH calculated from green:cyan fluorescence. Moreover, a critical benefit of the DsRed monomer (or any fused fluorophore not affected by pH or chloride) is that it provides an internal normalizer, so that variations in cell number, or magnitude of expression, from well to well do not produce variations in the signal ratio. This is an advantage over single-fluorophore sensors, which have been successfully used in high-throughput assays, but necessarily preclude ratiometric measurements (Haggie et al., 2018). Other major advantages of genetically encoded fluorophores include resistance to photobleaching, absence of permeation/loss across the plasma membrane, and synthesis by the cell rather than exogenous administration. Alternative methods generally employ small-molecule dyes that have transient residence in the cytosol, and must be washed out of the extracellular fluid before analysis (Chub et al., 2006; Haggie et al., 2016; Ikeuchi et al., 2018; Untiet et al., 2017). Modern multi-well plate reader type fluorometers come with many advantages over microscopy. For example, they can be used for automated kinetic assays, possess on-board temperature regulators, can measure multiple excitation and emission wavelength pairs over relatively short durations, and can be used to calculate average fluorescent signals within a confluent well, which minimizes the influence of artefactual signals that can be found in single-cell microscopy. Furthermore, a multi-well format is often necessary for high-throughput screening of potential ligands or substrates for transporters and receptors.

SLC26A3, or DRA (downregulated in adenoma), is a transporter expressed in mammals, including rodents and humans, that exchanges chloride for bicarbonate. Its predominant role appears to be in the colon, where loss of function leads to severe congenital chloride losing diarrhea (Höglund et al., 1996). However, SLC26A3 has been proposed to bear another role in pathophysiology, as deletion in mice decreased serum oxalate by 60% and 24 h urinary oxalate excretion by 70% (Freel et al., 2013). Oxalate is a component of approximately 80% of kidney stones, giving this simple divalent anion a major role in renal disease (Sakhaee, 2009). There is ongoing debate about the relevance of SLC26A3 to colonic oxalate absorption, especially in humans. One study found a significant, but modest (<50%), increase in oxalate absorption in *Xenopus laevis* oocytes expressing hSLC26A3 (Chernova et al., 2003) and investigators deemed the transport weak. However, it was not clear in the study if chloride, a substrate, and hence competitor, was excluded from the extracellular transport buffer. Moreover, in the aforementioned mouse study by Freel et al., the reduction in colonic mucosal to serosal flux of oxalate in Slc26a3 knockout mice was only 41%, despite a very clear influence of the transporter on urinary oxalate. SLC26A3 does not appear to be expressed in kidney, indicating that urinary oxalate was altered by a change in colonic absorption, and hence, the blood concentration. Therefore, the relevance of SLC26A3 to oxalate absorption cannot be fully determined, or ruled out, solely on *in vitro* evidence, as a 41% decrease in transport may be very clinically significant if hSLC26A3 is the sole carrier mediating colonic oxalate absorption. Indeed, this has been proposed (Whittamore and Hatch, 2017).

Chinese hamster ovary (CHO) cells are the most widely utilized mammalian cell type in the pharmaceutical industry for production of therapeutic proteins (Butler and Spearman, 2014). CHO cells are also widely used in the academic research setting. Their extensive use stems from their relatively simple handling requirements, suspension and adherent growth, simple medium, and their ability to assimilate and express foreign genes with protein glycosylation patterns similar to human (Butler and Spearman, 2014). The entire CHO cell genome has been sequenced and published (Dahodwala and Sharfstein, 2017). CHO cells can be engineered to stably and constitutively express genes, but are also amenable to inducible expression systems, such as various forms of tetracycline-on and tetracycline-off systems.

Here, we have employed CHO cells stably transfected with constitutively expressed ClopHensor, along with stably inserted tetracycline-inducible hSLC26A3 (SLC26A3-ClopHensor-CHO) to simultaneously determine the role of hSLC26A3 in oxalate transport, and gain some mechanistic insight about the strong endogenous oxalate transport function that we have discovered in our untransfected CHO cells. Employing these tools, we have achieved the following outcomes. (1) We confirmed that excellent chloride and pH standard curves could be generated with ClopHensor in a 96-well format, with pH-dependent chloride affinity values close to those reported using single-cell fluorescence microscopy. (2) We determined that live SLC26A3-ClopHensor-CHO cells could be effectively used to measure chloride transport and intracellular pH, and that bicarbonate exchange for chloride on SLC26A3 could be reliably and rapidly measured in this 96-well format. (3) We determined that an endogenous transport function mediating oxalate influx into CHO cells exists, and it is saturable, strong and sensitive to the inhibitor, niflumic acid. (4) We revealed that the endogenous oxalate transporter was unable to transport chloride, or specifically, was unable to exchange chloride for bicarbonate, unlike SLC26A3. The nature of the oxalate transport is intriguing, as niflumic acid is traditionally used to inhibit chloride transporters that, in some cases, also transport oxalate. In this case, CHO cells appear to express an oxalate transporter that is niflumate-sensitive, but that may not transport chloride. To date, all investigations on ClopHensor and derivatives (e.g. ClopHensorN) have used single cells with microscopy. Here, we report the successful application of ClopHensor in a 96-well assay using live adherent CHO cells.

## RESULTS

### hSLC26A3 expression and oxalate transport in CHO cells

**Fig. 1.**
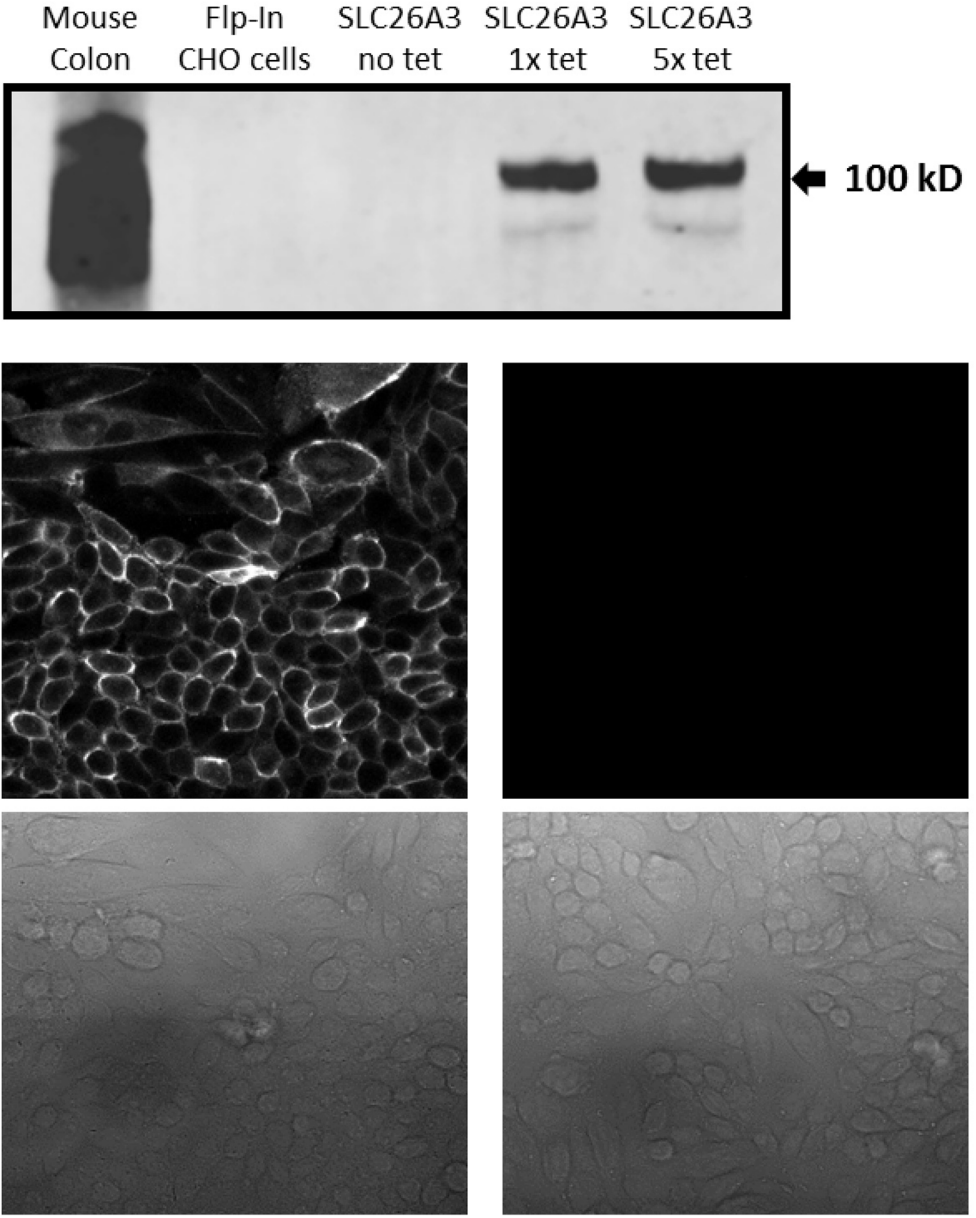
**Immunoblotting and immunofluorescence for SLC26A3 in CHO cells.** (Top panel) SLC26A3-CHO cells were induced with tetracycline 24 h before immunoblotting for human SLC26A3. Total protein from mouse colon was used as a positive control, Flp-In CHO cells and uninduced cells (no tet) were used as negative controls. Recommended and five times recommended tetracycline concentrations were tested (1× tet and 5× tet). Image capture on the LiCor Odyssey was set to default resolution and gain. (Middle panels) Induced (left) or uninduced (right) SLC26A3-CHO cells were incubated with the same primary antibody as in immunoblotting. Secondary antibody was AlexaFluor 488 anti-mouse. Bottom images are phase contrast light transmission of the cells in middle panels. No nuclear stain was used. Images at 400× magnification. The experiment was replicated more than three times over several months to ensure that expression (inducibility) was maintained in culture.

**Fig. 2.**
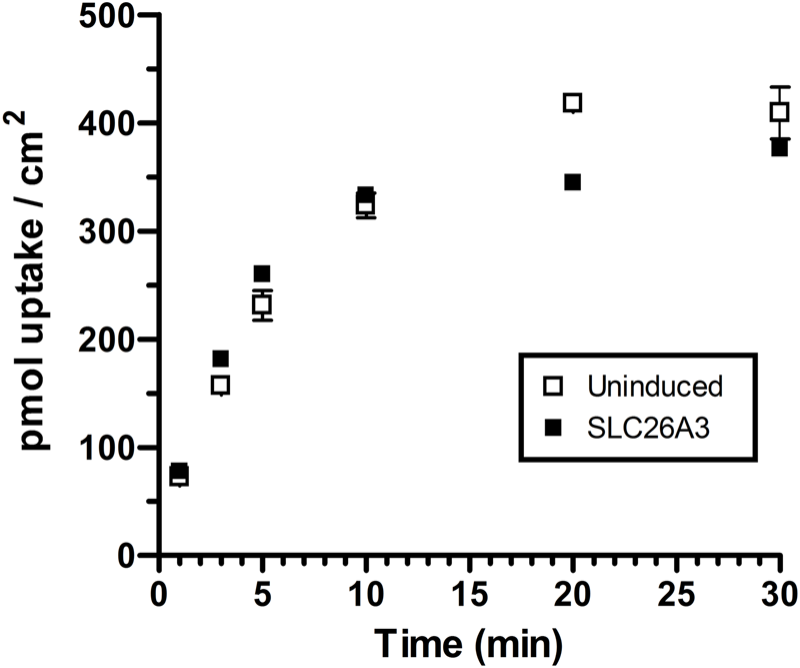
**Timecourse of oxalate uptake in uninduced and induced SLC26A3-ClopHensor-CHO cells.** Uninduced or 24-h tetracycline-induced cells were grown to confluence and incubated with ^14^C-oxalate at 100 µM at 1, 3, 5, 10, 20 and 30 min in chloride, calcium and magnesium-free buffer. Values are means±s.d. of triplicate measurements. No significant differences were observed at this concentration.

**Fig. 3.**
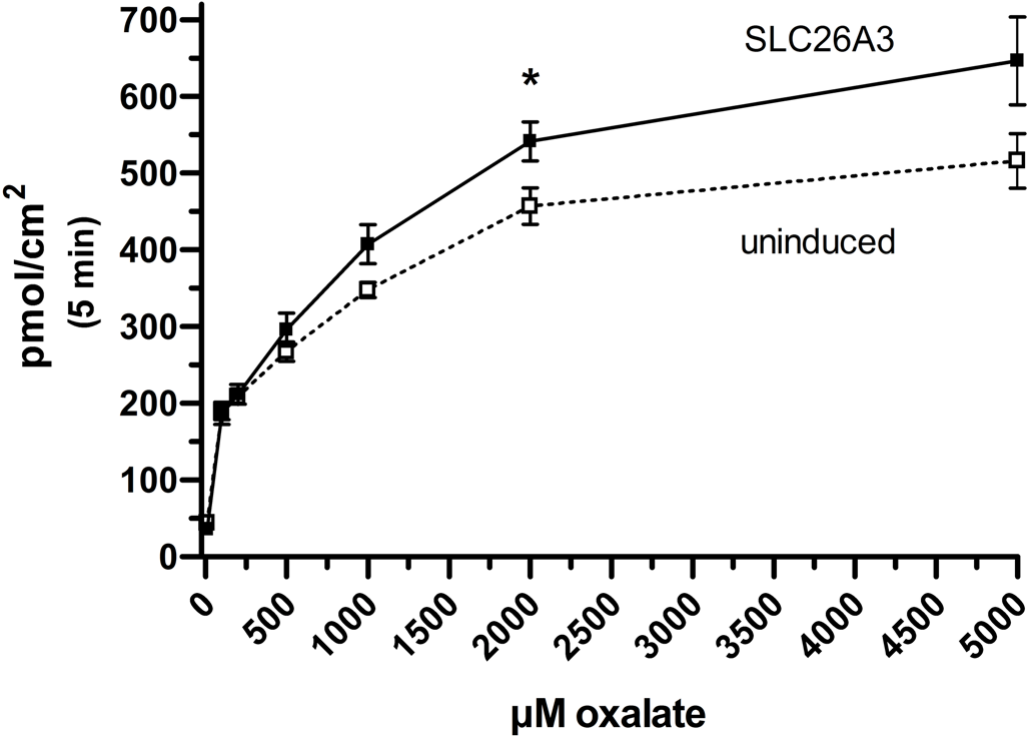
**Saturation of transport in uninduced and induced SLC26A3-ClopHensor-CHO cells.** Cells grown on 24-well plates were uninduced or tetracycline-induced 24 h before measuring transport of ^14^C-oxalate at room temperature for 5 min. *P<0.05 using two-tailed Student's *t*-test. Values are mean±s.d. of nine measurements (three triplicate experiments combined).

**Fig. 4.**
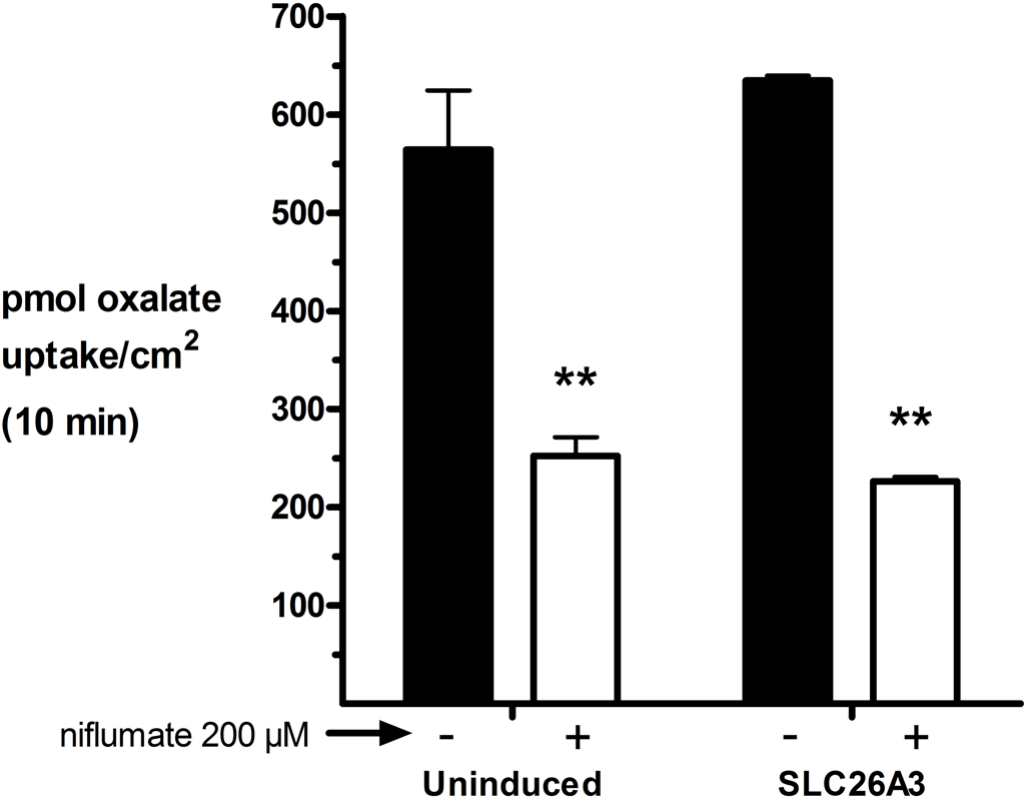
**Inhibitor-sensitive oxalate uptake in uninduced and induced SLC26A3-ClopHensor-CHO Cells.** Cells were induced with tetracycline 24 h before the experiment. Cells were incubated with 5 mM oxalate±200 µM niflumate (inhibitor) at room temperature for 10 min, washed three times and assayed by scintillation spectroscopy. Values are means±s.d. of three replicates. Representative experiment of three total is shown. **P<0.01 in two-tailed Student's *t*-test testing inhibitor effect.

This study was designed to determine the role of the human intestinal chloride transporter, SLC26A3, in oxalate transport, as the literature reports are inconclusive. We found that although SLC26A3 induction was successful and strong, and expression was membrane-localized, with no evidence of expression in uninduced cells (Fig. 1), oxalate uptake was no different at 100 µM (Fig. 2), and was only modestly greater than that in uninduced cells at higher concentrations (Figs 3 and 4). The greatest difference observed was at 5 mM oxalate, with statistical significance achieved only at 2 mM. However, it is very apparent in the saturation curve that CHO cells exhibit an endogenous oxalate transport activity that is concentration-dependent/saturable (Fig. 3). A chemical typically used as an inhibitor of chloride transporters, niflumate, was assessed for its ability to inhibit oxalate transport by SLC26A3, since this transporter has a major physiological role in absorbing chloride from the colon, and has previously been shown to be sensitive to niflumate. Niflumate inhibited oxalate transport substantially in both control cells and SLC26A3-expressing cells (Fig. 4), indicating that the endogenous CHO cell oxalate transporter is potentially also a chloride transporter. This hypothesis was tested, and results are presented in the next section.

### Chloride and bicarbonate transport in CHO cells

In order to determine the nature of the endogenous oxalate transporter in CHO cells, and specifically, to reveal whether it is truly also a chloride transporter, we compared control CHO cells with SLC26A3, the known chloride and bicarbonate exchanger in the colon. Given the dual intracellular changes anticipated with chloride and bicarbonate transport, i.e. pH modification with bicarbonate, and chloride concentration changes, we employed the dual-function biosensor, ClopHensor, which was originally tested and designed for assessing chloride and pH changes in neurons. All published studies on ClopHensor employed single cell fluorescence microscopy. In this study, we aimed to use a more automated method that was capable of measuring an average signal across a confluent well of cells, and directly measuring signal intensity, rather than utilizing image processing software. Moreover, the successful application of this transfected sensor in a multi-well viable cell assay will forge the way for investigators interested in high-throughput screening for chloride and pH modifying compounds without facing the limitations of exogenously administered dyes and time-consuming microscopy.

**Fig. 5.**
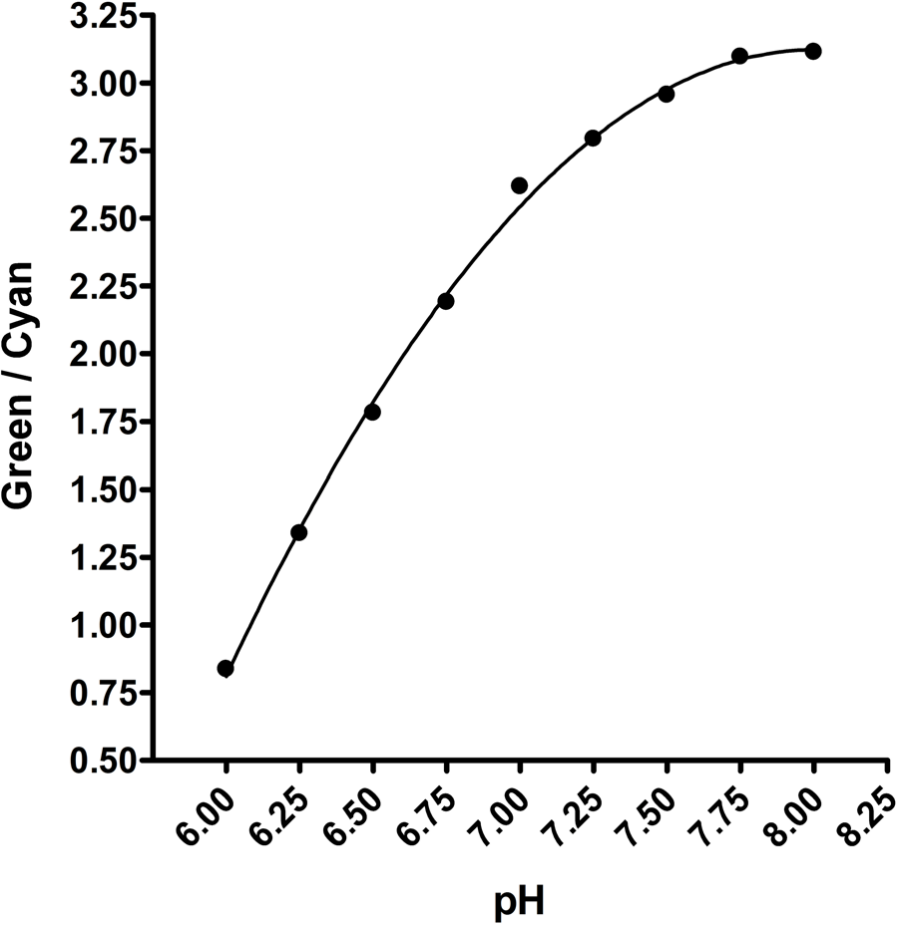
**pH standard curve in CHO cell lysate.** SLC26A3-ClopHensor-CHO cells were grown, pelleted, washed in zero chloride buffer and then lysed in ultrapure water. 5 µl aliquots were added to each well of a 96-well cover-glass bottom plate, and 95 µl of standard solutions were then added. Green/cyan fluorescence was measured and plotted using a second order polynomial equation on GraphPad Prism 4. Background fluorescence in control CHO cells was typically less than 5% of the signal intensity in ClopHensor-expressing cells (not shown), and when subtraction was performed, the curves were unaffected. Each data point is a mean of 21 measurements ±s.d. (three replicates at each chloride concentration makes up each data point). Error bars are obscured by data points.

**Fig. 6.**
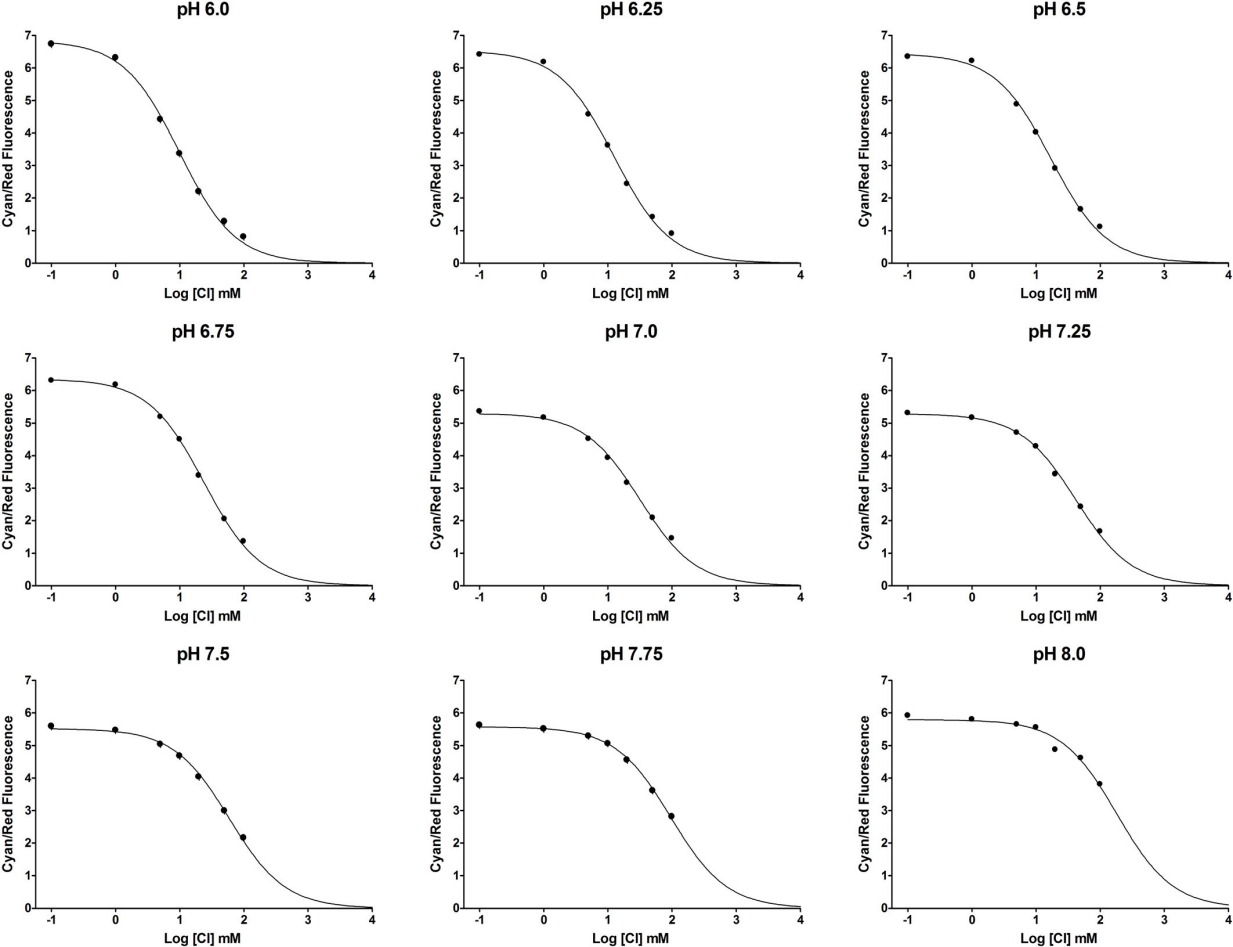
**Chloride standard curves in CHO cell lysate.** SLC26A3-ClopHensor-CHO cells were grown, pelleted, washed in zero chloride buffer and then lysed in ultrapure water. 5 µl aliquots were added to each well of a 96-well cover-glass bottom plate, and 95 µl of standard solutions were then added. Cyan/red fluorescence was measured and plotted using a ‘one-site competition’ model on GraphPad Prism 4. Curves were forced to end at zero in the software settings, consistent with the known complete quenching of cyan fluorescence when ClopHensor is 100% occupied by chloride. Background fluorescence in control CHO cells was typically less than 5% of the signal intensity in ClopHensor-expressing cells (not shown), and when subtraction was performed, the curves were unaffected. Each data point is a mean of triplicate measurements. s.d. bars are included for each value, but are obscured by the points. The experiment was repeated on three separate days over several weeks: first, with pH 6.0, 7.0 and 8.0, and then with pH 6.0, 6.5, 7.0, 7.5 and 8.0, and finally, the third experiment included pH 6.0, 6.25, 6.5, 6.75, 7.0, 7.25, 7.5, 7.75 and 8.0. Nine different pH curves were used in order to more accurately calculate intracellular pH in the chloride and bicarbonate exchange experiments.

**Fig. 7.**
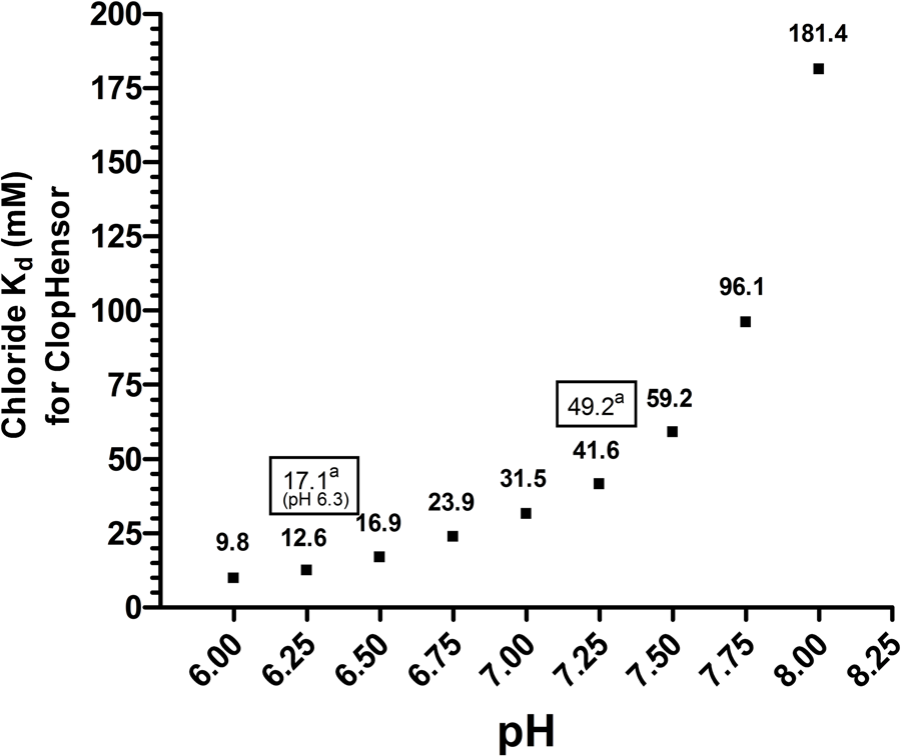
**pH-dependent chloride affinity for ClopHensor.** The K_d_ values using curves in Fig. 6 were calculated by GraphPad Prism 4 along with standard deviations. ^a^Values in boxes are from the original ClopHensor study (Arosio et al., 2010).

After several iterations and optimization steps to find the ideal plate type and plate reader settings, we produced a pH standard curve and nine chloride-pH standard curves in CHO cell lysate (Figs 5 and 6), due to the pH dependence of ClopHensor's chloride affinity. Although the standard curves were produced with cell lysate, the transport assays were performed in intact cells using the same plates. This was primarily to avoid the need for ionophores for permeabilizing the membranes when creating standard curves. We found that using cell lysate for curve generation yielded a pH-green/cyan fluorescence response (Fig. 5) and chloride (cyan/red fluorescence) affinity (K_d_) values (Fig. 7) that were very comparable to published values using live cells in microscopy. For this reason, we consider the extrapolation from lysed cells to live cells a valid approach. We found that this multi-well method yielded highly reproducible results, with error bars, in most cases, obscured by the data points.

**Fig. 8.**
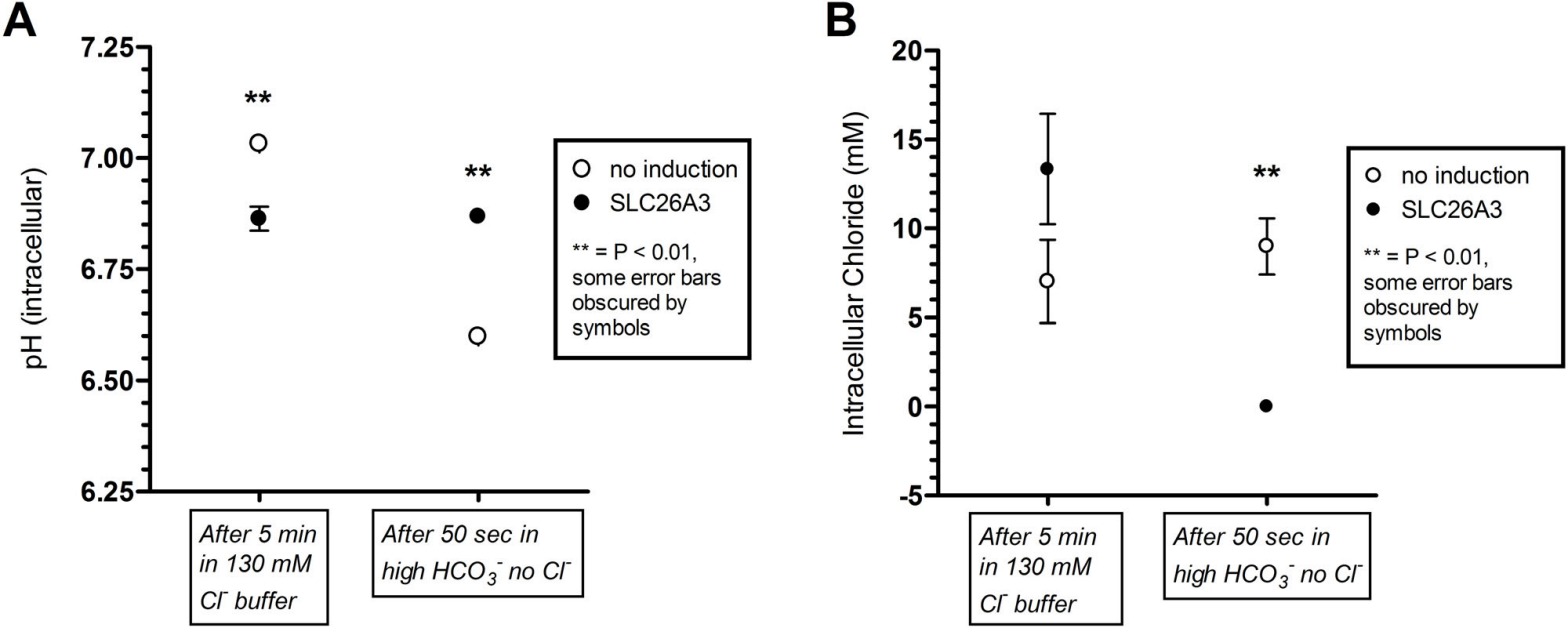
**Baseline pH and chloride and impact of high bicarbonate/no chloride in induced and uninduced SLC26A3-ClopHensor-CHO cells.** Cells were grown on cover-glass bottom 96-well plates to confluence. SLC26A3-induced and uninduced cells were compared at room temperature. (A) pH was calculated using ClopHensor green/cyan fluorescence ratios at baseline and 50 s after zero chloride/high bicarbonate was applied to the wells. (B) Chloride was calculated using cyan/red ratios also at baseline and after the same zero chloride/high bicarbonate conditions as in A. Data points are means of triplicate measurements±s.d. **P<0.01 in two-tailed Student's *t*-test comparing no induction versus SLC26A3. The experiment was performed three times in total with nearly identical results.

Using this optimized plate-based assay we determined that when cells were incubated with physiological extracellular chloride (130 mM) the SLC26A3-ClopHensor-CHO cells consistently maintained a higher concentration of intracellular chloride (Fig. 8B, most experiments achieved statistical significance). When subsequently incubated in the absence of extracellular chloride, and presence of excess bicarbonate, only the SLC26A3-ClopHensor-CHO cells demonstrated a very strong efflux of chloride (Fig. 8B). Although SLC26A3, as an exchanger, physiologically effluxes bicarbonate while taking in extracellular chloride, we exploited the ability of this transporter to work in the opposite direction (taking in bicarbonate while effluxing chloride). When pH was assessed after the application of zero chloride and high bicarbonate buffer, only the SLC26A3-ClopHensor-CHO cells were able to resist acidification, presumably by taking up bicarbonate (Fig. 8A). The mechanism of acidification by extracellular bicarbonate in a buffer maintained at physiological pH (7.4) in the control CHO cells has been described before, and will be revisited in the Discussion. Addition of niflumate in these fluorescence experiments was attempted, but consistently showed an influence on red fluorescence, either via the vehicle or the compound itself, confounding the interpretation of the ratiometric measurements. Therefore, these results are not presented.

## DISCUSSION

We report an advancement in the use of a dual-function biosensor, ClopHensor, constitutively expressed in CHO cells. Simultaneous intracellular chloride and pH measurement in a multi-well format yielded highly reproducible results and chloride affinity values comparable to those reported using microscopy. An encoded sensor provides several advantages over traditional dyes. (1) The sensor does not need to be added prior to the experiment in a time-sensitive manner, (2) the constant molar ratio of E^2^GFP to DsRed in this fusion protein allows for ratiometric measurements, therefore avoiding variable cell number, expression, or dye permeation as confounding factors, (3) long-term assays will not require re-application of dyes, thus preventing a significant source of variability, (4) technologies such as fluorescence activated cell sorting can be used to select for the highest expressers for clonal expansion, and (5) extraction and purification of the biosensor can allow for relatively inexpensive in-house use of this protein for extracellular pH or chloride assays. Transitioning from single-cell to multi-well assays is essential in developing a high-throughput screen. Considering the pivotal roles of chloride and pH in physiology and pathophysiology, and the routine use of CHO cells in industry, this system will likely lead to more rapid production of medications for diseases such as congenital chloride losing diarrhea, wherein SLC26A3 is nonfunctional in the colon, or constipation, and a number of other diseases. For instance, the GABA receptor/chloride channel in neurons is a major target for sedative drugs such as benzodiazepines and barbiturates, and anti-insomnia medications such as zolpidem. Furthermore, ethanol targets GABA channels, driving investigators to study alcohol withdrawal and addition treatments related to this receptor/channel. The system presented here can be employed with virtually any mammalian chloride channel or transporter in a high-throughput format.

The first biological question in this study (what is the role of human SLC26A3 in colonic oxalate absorption?) has not been conclusively answered. Instead, pursuit of this information has revealed that CHO cells possess a prominent endogenous oxalate transport function. This function is saturable, high-capacity relative to many transporters, and sensitive to niflumate. These are important findings for the following reasons: CHO cells are the most commonly employed mammalian cell line in therapeutic protein production and frequently used in academic research. Therefore, advancement in phenotypic characterization must parallel that in genotypic characterization. Oxalosis and hyperoxalemia research is pivotal in the fight against nephrolithiasis. In light of our findings, CHO cells may be deemed inappropriate for studying transporters that handle oxalate unless the endogenous transporter is identified and silenced/inhibited.

The second biological question (what is the nature of the endogenous oxalate transporter in CHO cells?) was generated from the above observations. We determined that the endogenous oxalate transporter, despite exhibiting sensitivity to niflumate, did not exchange chloride for bicarbonate (Fig. 8). That is, using SLC26A3-expressing cells as a positive control, we observed complete resistance to intracellular acidification only in cells expressing SLC26A3. Extracellular solutions at physiological pH (∼7.4) with high bicarbonate and another buffer source typically produce intracellular acidification (Levraut et al., 2001). Here, we used HEPES (10 mM) as the additional buffer. The acidification exclusively observed in uninduced cells indicates that they did not have a means to readily take in bicarbonate, unlike the SLC26A3-expressing cells. This finding, combined with the chloride efflux exclusive to SLC26A3-expressing cells, demonstrates that the endogenous oxalate transporter is not a typical bicarbonate/chloride exchanger. This is an important finding for the following reasons: the identification of a CHO cell transporter that handles oxalate, exhibits sensitivity to niflumate, but does not exchange chloride for bicarbonate, is a significant advancement, as it may represent a new carrier for oxalate. That is, niflumate is typically used to inhibit chloride transporters, some of which are also oxalate transporters (e.g. Slc26a6); however, to our knowledge, niflumate-sensitive oxalate transporters that do not handle chloride have not been reported. Accordingly, our future efforts will be directed towards identifying this transporter, and determining if CHO cell expression of this protein is an artificially evolved phenomenon (i.e. de-silencing of the gene in culture), or is reflective of expression in native ovarian tissue.

## MATERIALS AND METHODS

### Equipment and reagents

Flp-In CHO cells and the Flp-In T-Rex core kit containing the pcDNA5/FRT/TO plasmid and pcDNA6/TR plasmid were obtained from Invitrogen. The human SLC26A3 plasmid, HsCD00617533, was obtained from the DNASU Plasmid Repository (Arizona State University, Tempe, USA). Restriction enzymes, OneTaq DNA polymerase and Q5 DNA polymerase were obtained from New England Biolabs. SuperScript III and One-Step RT-PCR kits were purchased from Invitrogen. RNEASY kits were purchased from QIAGEN. Primers were ordered from IDTDNA. The pcDNA3-ClopHensor plasmid was obtained from Addgene nonprofit plasmid repository (Plasmid #25938). TransFectin^®^ Lipid Reagent was obtained from Bio-Rad (Hercules, USA). Lipofectamine 2000 was obtained from Thermo Fisher Scientific. Approved tetracycline-free serum was obtained from Clontech (now Takara Bio, Mountain View, USA). Ham's F-12 culture medium was obtained from Caisson Labs (Smithfield, USA). Hygromycin B was obtained from Toku-E (Bellingham, USA). G418 sulfate and sodium oxalate was obtained from Sigma-Aldrich (now MilliporeSigma). Blasticidin S was obtained from Thermo Fisher Scientific. 14C-oxalic acid was obtained from Moravek (Brea, USA) at 51 mCi/mmol and dissolved in ultrapure water. Ultima Gold liquid scintillation cocktail was obtained from Perkin Elmer (Waltham, USA). 96-well cover glass bottom black-wall tissue culture plates for fluorescence studies (cat# P96-1.5H-N) were obtained from Cellvis (Mountain View, USA). For fluorescence plate assays a SpectraMax^®^ M4 Plate Reader was used with SoftMax^®^Pro software. A Tri-Carb 2900TR Liquid Scintillation Analyzer was used for 14C-oxalate transport measurement. Immunoblots were analyzed on the Li-Cor Odyssey imager, and Odyssey Blocking Buffer and 2° antibody [IRDye 800CW goat anti-mouse IgG (H+L), 926-32210] were also obtained from Li-Cor (Lincoln, USA). NextGel 10% acrylamide and running buffer were obtained from VWR (Radnor, USA). Mouse monoclonal 1° antibody [SLC26A3 Antibody (H-8): sc-376187] was obtained from Santa Cruz Biotechnology. For immunofluorescence, an Alexa Fluor 488 goat anti-mouse IgG1 2° antibody was obtained from Thermo Fisher Scientific. VectaShield Anifade Mounting Medium was obtained from Vector Laboratories (Burlingame, USA). Imaging was performed on a Nikon Eclipse TE300 inverted microscope equipped with a short-arc mercury lamp (USH-102DH) from USHIO (Cypress, USA) and green fluorescence filter.

### Generation of SLC26A3-ClopHensor CHO cell line

Constitutive expression of SLC26A3 can result in loss of expression due to growth suppression. Therefore, we employed the Flp-In T-Rex system to produce tetracycline-inducible stably transfected SLC26A3 CHO cells. The pcDNA5/SLC26A3/TO construct was made by first amplifying the coding sequence of human SLC26A3 from plasmid HsCD00617533 with Q5 high-fidelity DNA polymerase using primers that added an Acc65i site immediately 5′ of the start codon and an XhoI site immediately 3′ of the stop codon. This amplicon was then cloned into the Acc65i and XhoI sites of pcDNA5/FRT/TO, creating pcDNA5/SLC26A3/TO. We maintained Flp-In CHO cells in zeocin-containing medium according to manufacturer recommendation, then plated the cells for transfection in 24-well plates, waiting for ∼50% confluence before transfection.

First, Flp-In CHO cells were transfected with the non-linearized pcDNA6/TR (tet-repressor) plasmid using TransFectin Lipid Reagent. Briefly, 100-µl aliquots of serum-free Hams F-12 medium were added to five sterile microcentrifuge tubes. To each of four tubes different amounts of purified pcDNA6/TR plasmid and TransFectin were added [1 µg plasmid/2 µl TransFectin, 3 µg plasmid/2 µl TransFectin, 1 µg DNA/4 µl TransFectin (most effective), and 3 µg DNA/4 µl TransFectin]. One tube contained only medium as a control. Each solution was mixed by pipetting, and incubated at room temperature for 20 min. Then, each solution was added to a different well of cells whose medium had been removed immediately before. Then 500 µl of serum-containing medium was added to each well, the plate was gently swirled to mix, then incubated at standard culture conditions for 6 h at which time another 500 µl of serum-containing medium were added to each well. At 24 h each well was lifted with 100 µl of trypsin (0.25% in PBS+1 mM EDTA) and transferred to another plate with fresh medium to distribute cells. The following day, medium was replaced with blasticidin-containing medium (10 µg/ml, determined from a kill curve to ensure killing of untransfected cells). The cells were grown for 3 weeks with blasticidin to allow selection and loss of transient transfection. After 3 weeks and substantial cell death, wells with viable colonies were lifted again with 100 µl trypsin, cells were counted and then diluted with medium to one cell/3 µl medium. 3 µl were then placed into the center of six separate dry wells of a fresh 24-well plate. This was repeated for each viable well from the transfection. All wells with a single cell were marked for continued growth as a clonal line. All wells with no cells or more than one cell were not used. The clonal lines were grown in blasticidin medium, and re-plated several times in the same well to distribute the colony. These resistant lines were grown up in flasks and used for RT-PCR of the tetracycline repressor transcript. RNA was extracted using an RNEASY kit and reverse-transcribed using a One-Step RT-PCR kit. The strongest expressing line was used for the next round of transfection with the pcDNA5/SLC26A3/TO plasmid. This transfection was essentially the same methodologically, but required the addition of the pOG44 plasmid in order to express the FLP recombinase for insertion of the transporter gene into the FRT site in the CHO cells. Various ratios of plasmid DNA to TransFectin were tried, ranging from 1–2 µg of SLC26A3 plasmid, and 4–20 µl of TransFectin, with 9 µg pOG44 plasmid in all tubes. Most conditions yielded at least some hygromycin-resistant cells (600 µg/ml), indicating successful plasmid entry into the cells. Initially nearly all cells were resistant, then over 2 weeks the vast majority died off. Survivor wells were assessed for SLC26A3 expression 24 h after induction with tetracycline. Unfortunately, only one of four survivor wells showed protein expression via immunoblot, which was weak. This well was used for dilutional subcloning as described with the pcDNA6/TR plasmid. Out of seven hygromycin-resistant clones, one showed very strong expression, three showed weak expression and the remainder were not inducible at all. The strong expresser was used for the third round of transfection (ClopHensor plasmid). The ClopHensor plasmid was transfected with a similar protocol, but 6 µg of plasmid DNA was mixed with 600 µl serum-free medium and aliquoted into two 300-µl portions. Either 4.5 or 12 µl Lipofectamine 2000 was added to these DNA-containing tubes, mixed and incubated for 10 min before adding 100-µl portions to wells, in triplicate, of cells grown to ∼50% confluence. 500 µl of serum-containing medium was added immediately after, and then cells were incubated for 24 h before G418 was added with fresh medium. Cells were maintained in G418 (and blasticidin and hygromycin) to select for constitutive ClopHensor expressers. After several weeks, dilutional subcloning was used to isolate highly-fluorescent clones as observed with a fluorescence microscope set at red or green fluorescence. Red fluorescence was more suitable for rapid assessment in culture due to the high background green fluorescence in the culture medium. The final triple transfected clones containing the tetracycline repressor, the tetracycline operator controlled SLC26A3 and ClopHensor were used for all fluorescence and transport experiments presented in this study.

### Immunoblotting

Confluent 75 cm^2^ flasks of uninduced or 24-h tetracycline-induced SLC26A3-ClopHensor-CHO cells were lifted with trypsin, washed three times with PBS to remove trypsin, and pelleted before aspiration of all supernatant. An approximately equal volume of ice-cold lysis buffer was added to the cell pellets (e.g. 25–30 µl), and cells were disrupted aggressively with an ultrasonic probe set to medium setting. To avoid substantial heating of the suspension, this sonication was performed with cells in ice, and was done in five separate bursts with 20 s in between each. Cells were then pelleted at 20,000× ***g*** at 4°C for 10 min. Precipitated SDS was sometimes visible after centrifugation, but did not compromise the results. Total protein was determined using the bicinchoninic acid (BCA) microplate method with samples diluted 25×. 100 µg of protein (diluted with lysis buffer if necessary) was mixed with 2× Laemmli loading dye containing beta-mercaptoethanol, and incubated at 37°C for 30 min. Boiling samples resulted in no signal on the blot, likely due to aggregation of lipophilic domains. Samples were separated on a NextGel 10% acrylamide gel at 100 V for 1.5 h, then transferred to a PVDF membrane at 20 V ice cold overnight. Blots were then blocked with Odyssey Blocking Buffer for 1 h at room temperature before overnight incubation with 1° antibody against SLC26A3 (1:100 dilution in Odyssey Blocking Buffer plus 0.1% Tween-20). After four 5-min washes in PBS+0.1% Tween-20, the membranes were then incubated in 2° antibody (1:5000 dilution in Odyssey Blocking Buffer plus 0.1% Tween-20) for 1 h at room temperature. After four 5-min washes in PBS+0.1% Tween-20, then one rinse with plain PBS, the blots were imaged on the Li-Cor Odyssey imager with default settings.

### Immunofluorescence

Uninduced or 24-h tetracycline-induced SLC26A3-ClopHensor-CHO cells were grown on #1.5 coverslips. Cells were rinsed twice with PBS, and immediately fixed with 4% paraformaldehyde (not formalin) in PBS for 10 min at room temperature. Cells were washed in PBS for 10 min three times, and were then permeabilized for 10 min in 0.1% Triton X-100 in PBS. Cells were then washed in PBS for 10 min three times. Cells were blocked for 60 min with 1% bovine serum albumin (BSA) in PBS, then incubated with the same 1° antibody used in immunoblotting (1:50 dilution in PBS, 1% BSA) in a humidified chamber overnight at 4°C. The next day cells were washed with PBS for 10 min three times. The secondary antibody (Alexa Fluor 488 anti-mouse, 1:400 dilution or 5 µg/ml in 1% BSA in PBS) was applied for 60 min at room temperature. Cells were washed with PBS for 10 min three times, and then mounted with VectaShield mounting medium. Cells were imaged using phase-contrast light transmission or green fluorescence.

### Oxalate transport in SLC26A3-ClopHensor-CHO and control CHO cells

For all oxalate transport experiments 24-well plates were seeded to reach confluence in 48 h. A day after seeding, or 24-h prior to the experiments, tetracycline was added to wells to induce SLC26A3. For all experiments, the cells were washed rapidly with transport buffer before applying transport buffer with oxalate. We avoided the typical 10 min pre-incubation with transport buffer in order to avoid loss of intracellular chloride (which may exchange for oxalate) and avoid the potential for cell lifting due to the calcium and magnesium-free nature of the buffer. The transport buffer contained sodium gluconate (130 mM), potassium gluconate (5.33 mM) potassium phosphate monobasic (KH_2_PO_4_, 0.44 mM), sodium phosphate dibasic (Na_2_HPO_4_, 0.34 mM), D-glucose (5.56 mM) and HEPES (10 mM). Chloride was excluded, as it is a substrate for SLC26A3. Calcium and magnesium were excluded as they precipitate oxalate immediately. pH was adjusted to 7.4 with sodium hydroxide. Hydrochloric acid was avoided, as it is a source of chloride, and the buffer was always acidic before base addition.

The general transport protocol is described here, while details are in figure legends. Cells were rapidly washed with transport buffer, then immediately incubated at room temperature with 300 µl transport buffer containing 14C-oxalic acid (typically 0.5 µCi/ml) and non-radiolabeled sodium oxalate. After the indicated times, the cells were rapidly washed with ice-cold transport buffer, then lysed with 0.2 M sodium hydroxide and repeated pipetting. The entire lysate (200 µl) was transferred to scintillation vials with Ultima Gold scintillation cocktail (4 ml) and counted. For inhibition with niflumate, niflumic acid was dissolved in DMSO and the concentrated stock was added to complete transport buffer to yield a 200 µM solution. Niflumic acid precipitated and adhered to plastic tubes before it could be completed distributed in the buffer. Therefore, glass vials were used for preparing niflumate-containing solutions.

### Generation of chloride and pH standard curves with ClopHensor

For chloride and pH standard curves we grew SLC26A3-ClopHensor-CHO cells in 225 cm^2^ flasks to confluence, lifted the cells with trypsin, pelleted, washed and pelleted three times with 5 ml of chloride-free buffer (transport buffer above), and after final pelleting, lysed in a small volume of water. Since the lysate had a more diluted ClopHensor protein than intact cells, we generally used the entire 225 cm^2^ flask for a 96-well plate. For example, a cell pellet from a 225 cm^2^ flask was lysed in 500 µl of ultrapure water with an ultrasonic probe. The lysate was centrifuged at 20,000× ***g*** for 10 min, and 5 µl of supernatant was applied to each well of the cover glass-bottom 96-well plate with black walls. Then, 95 µl of chloride and pH standard buffers were added to the wells and mixed. The 96-well plates were read on the SpectraMax M4 plate reader at the following wavelengths: green fluorescence at 488 ex./525 em., cyan fluorescence at 458 ex./525 em., and red fluorescence at 543 ex./600 em. Each well was scanned at nine evenly distributed points and averaged automatically with the software. The chloride and pH standards were formulated as follows: all solutions started from the same stock containing calcium gluconate (1.26 mM), magnesium gluconate (0.897 mM), potassium gluconate (5.33 mM), potassium phosphate monobasic (0.441 mM), sodium bicarbonate (4.167 mM), sodium phosphate dibasic (0.338 mM), glucose (5.56 mM), HEPES (10 mM) and citric acid (10 mM). The citric acid was used to buffer in the lower pH range, whereas HEPES was for buffering in the higher pH range. This stock solution was aliquoted to create a range of chloride concentrations. For example the 0 chloride solution was stock plus 137.93 mM sodium gluconate, the 50 mM chloride solution was stock plus 50 mM sodium chloride and 87.93 mM sodium gluconate. In each case, the balance of electrolyte was provided by sodium gluconate. Each of these solutions were then further aliquoted to create solutions of various pH values. We used citric acid and sodium hydroxide for pH modification to avoid additional chloride from HCl.

As expected, the green/cyan fluorescence was sensitive to pH, whereas the cyan/red fluorescence was sensitive to chloride. As reported previously, because both green and cyan fluorescence are affected equally by chloride, their ratio does not change significantly when chloride is altered, but pH is maintained. The standard curves were generated on GraphPad Prism 4 by GraphPad Software (La Jolla, USA) using a one-site binding curve fit for chloride and a second order polynomial fit for pH.

### Chloride and bicarbonate transport in live SLC26A3-ClopHensor-CHO cells

For live cell experiments the same glass-bottom tissue culture plates that were used for standard curves were employed. At 48 h prior to transport experiments cells were plated into each well of the 96-well plate. Rather than using cell counting, we used surface area to calculate an estimated time to confluence. For example, each well in the glass-bottom 96-well plate is 0.3 cm^2^ and cells were grown in 25 cm^2^ flasks. Adherent CHO cells have a lag phase for about 24 h and then double about every 17 h thereafter. Therefore, we plated enough cells into each well so that one doubling would give confluency (i.e. <48 h after plating cells would be confluent). Typically, we resuspended a pellet obtained from a confluent 25 cm^2^ flask and plated 0.6% of the cells (0.6% of the resuspended volume) in 200 µl for each well. The importance of cell density for the time of experiment should not be overlooked. If cells are too confluent they detach, whereas low confluency results in low fluorescent signal. Tetracycline was added to half the wells, with fresh medium, at twice the recommended concentration for induction 24 h before the experiment.

The dual function of ClopHensor allowed simultaneous measurement of intracellular pH and chloride at each step in the transport experiment. Two different buffers were used for the experiment, both starting from the same stock solution: stock=calcium gluconate 1.26 mM, magnesium gluconate 0.49 mM, magnesium sulfate 0.41 mM, potassium gluconate 5.33 mM, potassium phosphate monobasic 0.44 mM, sodium phosphate dibasic 0.34 mM, D-glucose 5.56 mM, HEPES 10 mM and sodium bicarbonate 4.17 mM; solution A=stock+130 mM sodium chloride; solution B=stock+50 mM sodium bicarbonate and 80 mM sodium gluconate. Solution B was devoid of chloride and had excess bicarbonate for driving exchange on the transporter with intracellular chloride. Both solution A and solution B were adjusted to pH 7.4 with sodium hydroxide, and no hydrochloric acid was used.

For the experiment, the medium was removed, cells were washed twice with 200 µl of solution A, incubated at room temperature for 5 min, then scanned at the green and cyan wavelengths at room temperature. Solution A was replaced with 200 µl of solution B, then the plate was scanned again at 50 s for green and cyan fluorescence. Finally, the plate was scanned for red fluorescence in order to allow for ratiometric calculation of chloride concentration. Red fluorescence was scanned at the end to allow more rapid measurement of green and cyan during the experiment, as the RFP in ClopHensor is not affected by pH or chloride and serves an ideal method for normalizing to cell number and ClopHensor expression. That is, when using dyes, variations in cell number will create variability in the signal intensity. In ratiometric measurements, this source of error is inherently eliminated. In processing the raw data, the intracellular pH was calculated first in order to determine the appropriate chloride standard curve to use for chloride calculation. This is due to the pH dependence of chloride affinity for ClopHensor, and hence the altered absolute cyan fluorescence for a given concentration of chloride when pH is modified. All interpolations on the standard curves were performed in GraphPad Prism 4 using the ‘unknowns from standard curve’ function. The chloride affinity (dissociation constant, or K_d_) was represented as the EC_50_ on the software, as discussed in the user manual for the program.
